# Clinical and Translational Science Personas: Expansion and use cases

**DOI:** 10.1017/cts.2023.572

**Published:** 2023-06-08

**Authors:** Sara Gonzales, Robin Champieux, Nicole Contaxis, Andrea H. Denton, Mohammad Hosseini, Alisa Surkis, Annie Wescott, Kristi Holmes

**Affiliations:** 1 Galter Health Sciences Library and Learning Center, Northwestern University Feinberg School of Medicine, Chicago, IL, USA; 2 OHSU Library, Oregon Health and Science University, Portland, OR, USA; 3 Health Sciences Library, NYU Langone Health, New York, NY, USA; 4 Claude Moore Health Sciences Library, University of Virginia, Charlottesville, VA, USA; 5 Department of Preventive Medicine, Northwestern University Feinberg School of Medicine, Chicago, IL, USA

**Keywords:** Methods and processes, translational workforce, implementation, interprofessional team, technology, patient perspectives, persona, qualitative, user-centered design, organizational and social issues

## Abstract

Twelve evidence-based profiles of roles across the Clinical and Translational Science (CTS) workforce and two patient profiles were developed by CTS Personas collaborators in 2019 as part of the CTSA Program National Center for Data to Health (CD2H). Based on feedback received from the community, CTS Personas team members collaborated to produce five additional Personas to broaden representation of the CTS workforce and enhance the existing portfolio. This paper presents the rationale and methodology used in the latest CTS Personas initiative. This work also includes an implementation scenario incorporating multiple Personas. Using the new National Institutes of Health’s (NIH) Data Management and Sharing Policy as an example, we demonstrate how administrators, researchers, support staff, and all CTS collaborators can use the Personas to respond to this new policy while considering the needs of service providers and users, CTS employees with short- and long-term needs, and interdisciplinary perspectives.

## Background

### The CTS Personas Evolution and New Releases

The Clinical and Translational Science (CTS) Personas [1] were developed and shared with the community in 2019 as part of a collaborative project of the Clinical and Translational Science Awards (CTSA) Program National Center for Data to Health (CD2H, Grant U24TR002306). The CTS Personas project aimed to produce representative profiles of people working and interacting within the field of translational science, to present evidence-based portrayals of their needs, motivations, goals, and pain points. The goal of developing this portfolio of Personas was to provide representative profiles of translational science staff to inform the development of software solutions, educational and communication materials, and other professional resources. Leveraging workflows and methodologies developed by various business, academic, and federal government institutions as a blueprint [2–4], team members from Northwestern University’s Feinberg School of Medicine, Children’s Hospital of Philadelphia, Oregon Health & Science University (OHSU), and Washington University came together to develop the CTS Persona profiles.

**Figure 1.**
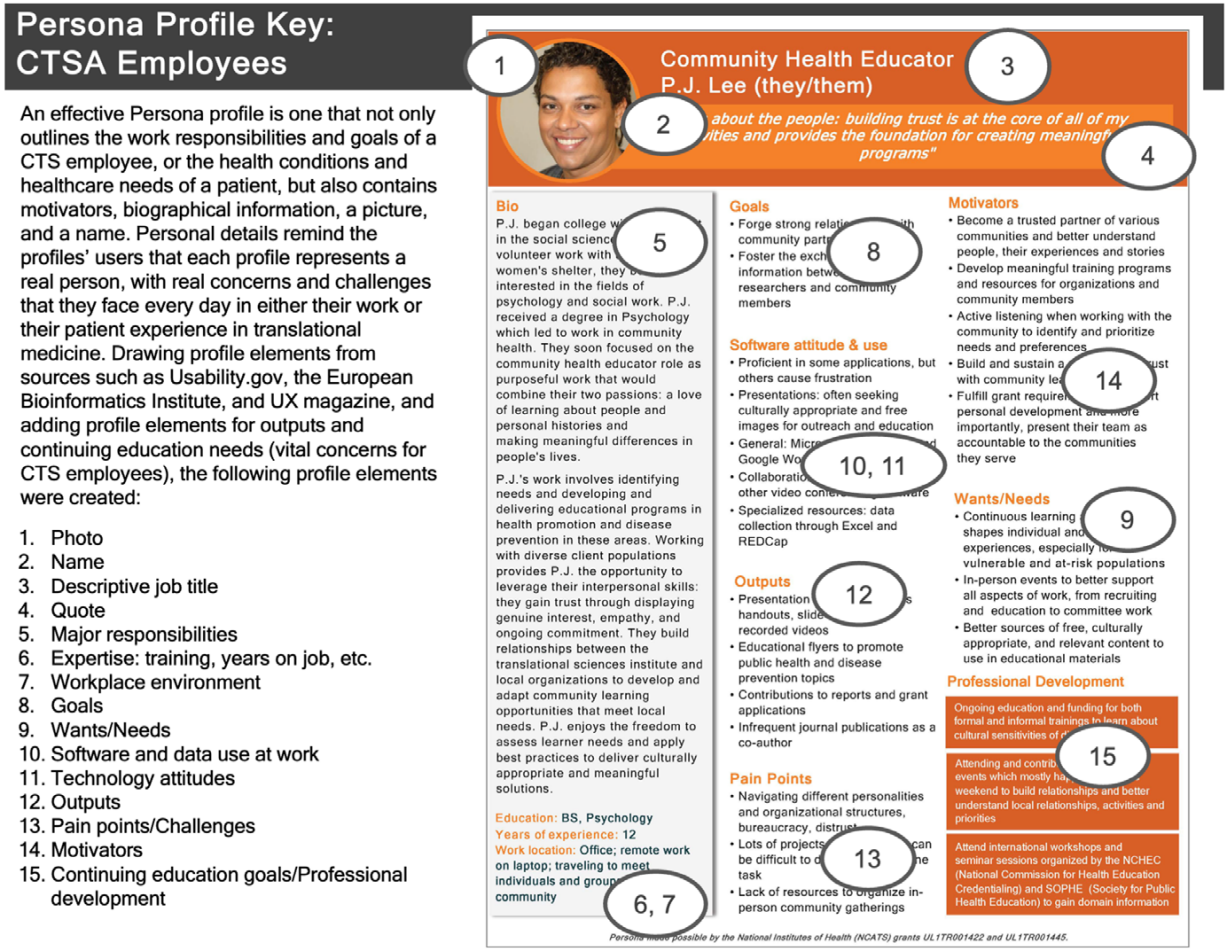
A key to the elements comprising each of the CTS Personas, with each element numbered on the template.

Our methodology in 2019 [5] involved three phases:

- Identify key CTS roles
- Identify required elements for outlining Personas (Fig. 1)
- Develop the Persona profiles based on information retrieved from literature searches and interviews

**Figure 2.**
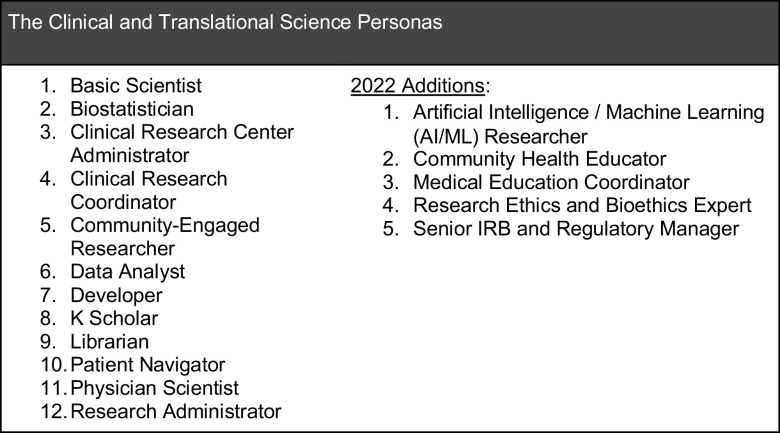
The original twelve CTS workforce Persona profiles, plus the five additions for 2022.

Through the original project, we identified one dozen CTS roles to profile and developed a 15-element Persona profile template incorporating CTS workforce characteristics such as professional needs, goals, challenges, software use, and outputs [5]. Our final product was a portfolio of 14 Persona profiles, including 12 CTS employee profiles and 2 patient profiles (Fig. 2).

After a successful rollout and adoption of the CTS Personas by the CTSA community [6], the team received requests from community members to develop additional Persona profiles to complement the original portfolio. Several members of the first project team committed to participate in the expansion and were joined by colleagues from additional institutions. The 2022 project team included team members from Galter Health Sciences Library at Northwestern University Feinberg School of Medicine, Oregon Health & Science University (OHSU) Library, the Claude Moore Health Sciences Library at the University of Virginia, and the New York University Health Sciences Library.

## Methods

The team collated the suggested Persona profiles from the community and discussed other potential additions. We created a REDCap poll asking CTS employees to vote on their top choices among the proposed Personas and made it available through the Center for Leading Innovation and Collaboration (CLIC), a key service organization of the CTSA community. We aimed to identify the five most highly ranked roles, which we would then profile using our established methods. Toward this end, we employed the same template of elements that was used to describe the 2019 Personas. We also used the same methodology to collect and analyze three to five interviews and literature/website reviews for each Persona. Between October 2021 and August 2022, we developed five additional CTS Personas profiles [7]:

- Artificial Intelligence/Machine Learning (AI/ML) Researcher
- Community Health Educator
- Medical Education Coordinator
- Research Ethics and Bioethics Expert
- Senior IRB and Regulatory Manager

The addition of five new profiles inspired the team to re-examine the previous profiles from a viewpoint of continuous improvement and inclusion. The new portfolio features an upgraded design and color coding scheme, where the background color denotes the Persona’s position in the NIH-outlined spectrum of translational science [8]:

- Lavender: Basic Research
- Blue: Pre-Clinical Research
- Green: Clinical Research
- Yellow: Clinical Implementation
- Orange: Public Health
- Red: Patients
- Gray: Role Spans the Translational Workforce

Similarly, decorative but potentially distracting icons, as seen in the 2019 profiles, have been removed. Software use described in the Personas was classified by type (e.g., General, Communication, and Specialized Resources) to ensure the continuity of themes, even as specific tools may change over time. In addition, gender-neutral pronouns have been incorporated for two of the profiles to reflect a more inclusive and equitable representation of the CTS workforce and community. The CTS Personas team believes that these design updates make for a leaner and more user-friendly profile template.

## Discussion

### An Implementation Scenario Incorporating Multiple Persona Use Cases

As with the 2019 portfolio, these new Personas are intended to be used in brainstorming, role-playing, and proof-of-concept exercises by members of the CTSA community. Persona profiles can also be useful in providing more accurate and inclusive language to promote CTS activities, developing software and other resources, and creating relevant and useful educational resources. When planning process improvements or changes, we encourage institutions to select the Persona profile[s] that most closely match the participants in their scenarios and walk through specific interactions and solutions. The Personas can help institutions approach challenges from multiple perspectives, such as those of both service providers and service users. Furthermore, the Personas’ individual characteristics can encourage those utilizing them to consider different CTSA employees’ unique needs and concerns. To demonstrate such application of the CTS Personas, we developed the following scenario describing how an updated federal policy sparks challenges, needs, and solutions that involve and affect many workforce members at a CTSA institution. The provided scenario helps demonstrate how CTSA leadership could consult the CTS Personas in order to better understand involved stakeholders, assign duties, provide guidance, and facilitate partnerships.

### NIH Data Management and Sharing Policy Compliance: An Institutional Scenario

**Figure 3.**
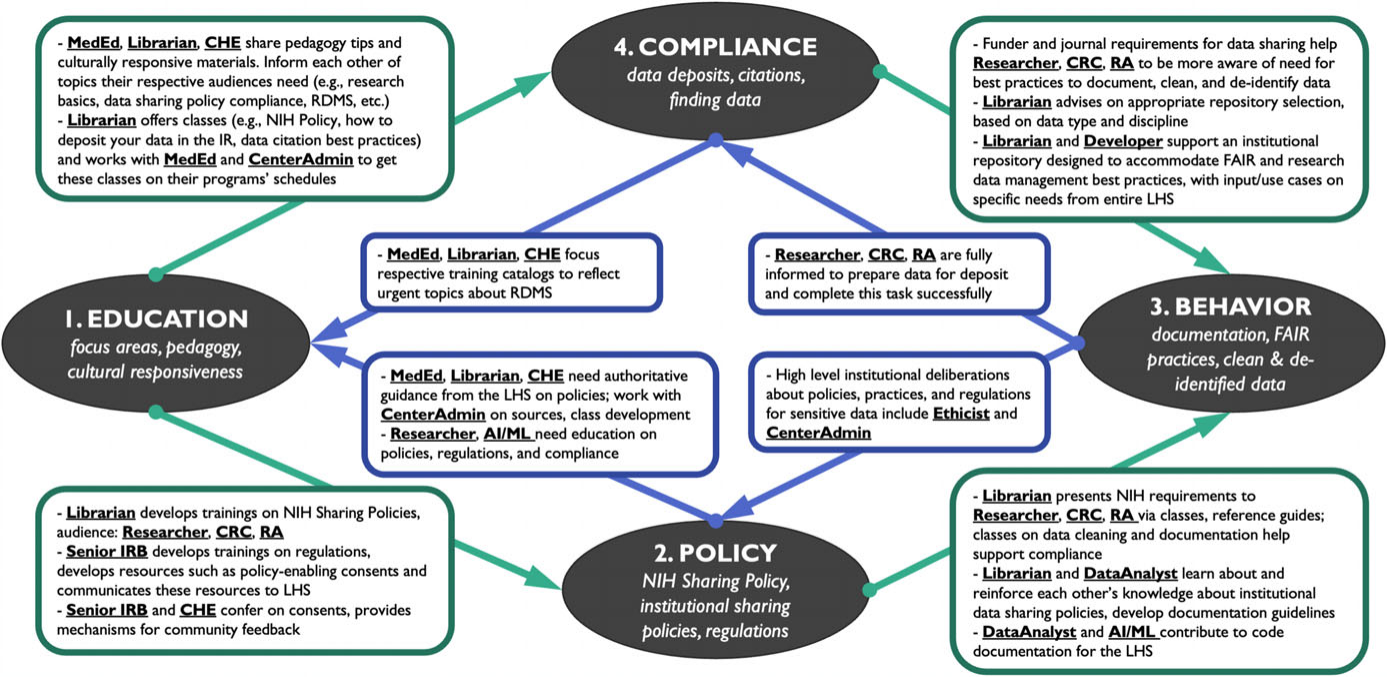
The interrelated activities of a learning health system (LHS) adapting to the new NIH Data Management and Sharing Policy necessitate behavioral adjustments, which are fostered through education, to promote compliance. LHS roles, depicted by the CTS Personas, highlight new aspects of essential knowledge and behaviors that should be considered in creating educational resources and new approaches to their work. Education, policy, behavior, and compliance are numbered 1–4, respectively, in the diagram, corresponding to four subsections below detailing the use case. **Abbreviation Key: AI/ML**, Artificial Intelligence/Machine Learning Researcher Persona profile; **CenterAdmin**, Clinical Research Center Administrator Persona profile; **CHE**, Community Health Educator Persona profile; **CRC**, Clinical Research Coordinator Persona profile; **DataAnalyst**, Data Analyst Persona profile; **Developer**, Developer Persona profile; **Ethicist**, Research Ethics and Bioethics Expert Persona profile; **Librarian**, Librarian Persona profile; **LHS**, Learning Health System; **MedEd**, Medical Education Coordinator Persona profile; **Researcher**, includes all Researcher Persona profiles: Basic Scientist, Community-Engaged Researcher, K Scholar, Physician Scientist, Research Ethics and Bioethics Expert, Artificial Intelligence/Machine Learning Researcher; **RA**, Research Administrator Persona profile; **Senior IRB**, Senior IRB and Regulatory Manager Persona profile.

The National Institutes of Health’s (NIH) Data Management and Sharing Policy (NIH DMS Policy) went into effect on January 25, 2023 [9]. This policy requires that all NIH grant applications include a data management and sharing plan that delineates data management strategies throughout the project life cycle, as well as how the data will be shared at the end of the project or upon publication. While data sharing has been standard practice in some CTSA disciplines, for many researchers this practice is new and requires rapid knowledge acquisition and skill development regarding data policies, research data management best practices, and repositories for sharing data (Fig. 3).

The launch of the NIH DMS Policy catalyzes an immediate need for awareness and understanding at a CTSA institution (learning health system or LHS) regarding the policy’s requirements, institutional data sharing policies, and regulations that govern the responsible collection and sharing of data. For such information, researchers, lab coordinators, and administrators rely on training and online resources offered by research support professionals such as Librarians, Senior IRB and Regulatory Managers, the Office of Research, and Medical Education Coordinators. However, different Researchers (e.g., Physician Scientists, Community-Engaged Researchers, K Scholars, and Artificial Intelligence/Machine Learning Researchers) will have unique educational needs based on specific factors such as software attitude and use, outputs and pain points. Using Persona profiles developed for different groups of Researchers can increase the effectiveness of educational materials. Aligning educational practices with policies while serving a variety of Personas is a first step in process improvements, which will lead to behavioral adjustments and full NIH DMS Policy compliance, as outlined below. In each of these steps, employment of the Personas demonstrates how staff in various roles both contribute to change and benefit from it.

### Education: Focus Areas, Pedagogy, and Cultural Responsiveness

Education about the NIH DMS Policy and institutional data policies is a key first step to support compliance, and the Librarian and Medical Education Coordinator Personas are on the education front line. The Librarian’s profile mentions expertise in research impact and bioinformatics. She takes on additional responsibility to become fluent in relation to the NIH DMS Policy, as it is closely related to this work, and LHS has no plans yet to hire a data librarian. The Librarian develops a class on the NIH DMS Policy, conferring with the Medical Education Coordinator on pedagogy and instructional design to ensure an effective training opportunity. These two Personas also consult the Community Health Educator for their expertise on culturally responsive and supportive resources to make the class materials inclusive and accessible. As these Personas share their skills, and resources to support NIH DMS Policy compliance are developed, it becomes clear that these classes must be offered as part of the continuing medical education and CTSA administration curricula at LHS. The three content developers consult with the CTSA Center Administrator, who assesses the new materials against existing educational offerings and the Center’s schedule, in order to incorporate these vital new classes.

### Building Policy Awareness: NIH Data Sharing Policy, Institutional Sharing Policies, and Regulations

Numerous existing institutional policies and workflows have an impact on NIH DMS Policy compliance, and Researchers, Clinical Research Coordinators, and Research Administrators need to be kept up to date on the latest guidelines. In addition to consulting the Office of Research and federal and state sources on policies, Clinical Research Coordinators and Research Administrators, as well as the Librarian, Medical Education Coordinator, and Community Health Educator can benefit from trainings and resources developed and offered by LHS’s Institutional Review Board (IRB) office, overseen by the Senior IRB and Regulatory Manager. In addition, a key aspect of compliance with the NIH DMS Policy is that research participants should be fully informed during the consent process to the broad sharing of data beyond the original project for which it was collected. The Research Ethics and Bioethics Expert outlines the conditions of consent and potential ethical issues in relation to collection, use, storage and retention of data, and highlights the need to update LHS’s consent templates. Toward this end, the Senior IRB and Regulatory Manager works with the Community Health Educator to ensure that consent forms comply with the NIH DMS Policy while also being understandable for research participants. The Community Health Educator could pilot drafted consent forms with the community for feedback to ensure that future participants fully understand the content as well as their rights (e.g., to disagree with data reuse) as study participants.

### Best Practice Behaviors: Data Documentation, FAIR Practices, and Clean and De-identified Data

Beyond being aware of NIH Policy and having up-to-date research protocols and consent templates, both researchers and research support professionals require assistance in crafting effective data management and sharing plans. They also need guidance to proficiently clean and curate their data, and to make the data available in a FAIR (Findable, Accessible, Interoperable, and Reusable) manner [10]. For example, because openness of anonymized data might sometimes make participants vulnerable [11], institutional conversations must involve decision-makers and the Research Ethics and Bioethics Expert, CTSA Center Administrator, and Researchers throughout the institution to ensure that data security and compliance are balanced without compromising participants’ interests. Data sharing and security policies developed as a result of these conversations will have a direct bearing on institutional data management best practices across LHS.

Informed by internal and external regulatory and data policy requirements, data management best practice classes and resources can be developed by the Librarian. Classes and LibGuides can offer training from beginner to advanced levels in data cleaning, documentation, and making data FAIR. Classes are designed with various Personas’ needs in mind, including LHS’s various Researchers, Clinical Research Coordinators, and Research Administrators. For example, since Microsoft Excel is often utilized for data cleaning, proficiency is noted in the profiles of the Clinical Research Coordinator and the Research Administrator, and this tool is included in relevant instructional materials. For more complex aspects of data documentation, the Librarian partners with the Data Analyst to learn best practices. Both have received instruction about institutional data sharing policies and have learned ways to document sensitive data. As a result of attending tailored classes, Researchers also learn about their limitations and ways to complement their skills. For example, while data de-identification is beyond the Librarian’s, Data Analyst’s, and most Researchers’ skills, they recognize and note when significant, difficult-to-remove personal identifiers are present in their data and know when and who to consult for further assistance. Upon submitting requests for de-identification assistance to the local biostatistics center, the Biostatistician assesses whether the center has in-house resources, or whether outside experts must be hired to complete the de-identification.

As the NIH DMS Policy spurs greater sharing of data, documentation and sharing of analytical code will also become increasingly common. The Data Analyst and AI/ML Researcher are both skilled in code documentation, with the AI/ML Researcher additionally proficient in metadata management and ensuring research reproducibility. They confer with the Medical Education Coordinator and Center Administrator to assess training needs on these topics in their curricula and add classes and supporting materials accordingly.

### Data Sharing Compliance Maturity: Data Deposits, Data Citations, and Finding Relevant Data

Best practice training and support for NIH DMS Policy compliance must also include support for sharing data through a digital repository by the end of the performance period or upon publication. With the end goal of data deposit in mind, Researchers and research support professionals, including Clinical Research Coordinators and Research Administrators, acknowledge the need to develop skills in data management, documentation, cleaning, and preparation for depositing data. They can negotiate with their respective continuing education professional associations to add training events on these topics to their certification curricula. As their skills grow, they can take advantage of local research data management refresher classes taught by the Librarian or those offered by the Medical Education department and encourage new hires to begin with these courses. Over time, their evolving data management skills will pave the way for improved long-term data practices, leading to more reproducible studies, a decrease in retractions, and enhanced data sharing and reuse through suitable repositories.

The Librarian’s networks and partnerships allow her to offer tailored data repository support, including repository selection, based on data type and discipline. She can guide Researchers toward NIH-supported repositories [12], as well as acceptable repositories in the Generalist Repository Ecosystem Initiative (GREI) [13]. At the LHS, the library administers an institutional repository, wherein the Librarian plays a curator role. The Librarian liaises with Researchers and support staff to catalog descriptive metadata about data deposits (e.g., creators, title, subjects, etc.) in the repository record. The Librarian can also assist with uploading research data. Such work is supported through strong relationship building with the Researcher and support staff communities. Due to potential time constraints, the Librarian may not be able to directly engage with these communities. However, by consulting the Researcher and support staff Persona profiles, she can gain insights into the specific needs, expertise, and data proficiency of these communities.

The LHS’s library owns and operates an institutional repository administered by the Librarian. The Developer persona in this case works in the library, updating the institutional repository’s code base and adding new features and modifications as necessary in order to meet the LHS community’s needs. The Developer values the chance to interact with subject matter experts at LHS and conducts informal interviews with them to learn about their specific use cases and needs from a repository tool. He then utilizes a collaborative, open, ticket-based system in GitHub to communicate about the requests and implement the required enhancements. The Librarian works with the Developer to ensure the data model and API (application programming interface, a machine-based way to access the repository’s data) of the institutional repository support data that are FAIR for humans and machines.

One of the Librarian’s focus areas is research impact. As data deposits grow, the Librarian is aware of evolving data citation initiatives [14] and can provide consultation services or classes to support data citation, impact assessment of deposited datasets, and identification of shared data for reuse for secondary analyses. By supporting the management, curation, deposit, and finding of data all the way through the life cycle of data gathering and reuse, the Librarian and all the LHS collaborators involved in this process benefit from the requirements of the NIH DMS Policy. LHS professionals represented by the Personas have increased their own knowledge by developing or attending trainings, by learning about the needs of their colleagues in LHS and often directly partnering with them, and in some cases by identifying and analyzing deposited data. The cycle of work to support the NIH DMS Policy, and what the faculty and staff have learned about each other along the way, either by direct partnership or by consulting their Persona profiles, has potentially made LHS a stronger institution.

The implementation scenario discussed above illustrates how skills, knowledge, and characteristics presented in the CTS Personas can inform workflows and best practices at a CTSA institution. We encourage readers to apply the CTS Personas to their own local scenarios and use cases. When using the library of Persona profiles, choose those that most closely represent the actors in your implementation scenarios, and walk these Personas through the steps of possible solutions with the following in mind:

- Consider both providers and recipients of a service or resource
- Consider immediate and long-term outcomes
- Proactively consider collaborative and interdisciplinary contexts
- Proactively incorporate ethics and equity

## Conclusion

### Concluding Remarks and Future Perspectives

As the above implementation scenario demonstrates, when planning process improvements or changes, the CTS Personas portfolio can be applied to elucidate a variety of professional requirements and perspectives. This project can continue to be relevant as the CTS Personas team considers evolving professional roles, additional profiles, inclusivity, and updates to the template design to improve accessibility and enhance coverage of the workforce.

Emerging roles in the CTS workforce are candidates for future updates to the CTS Personas. One such role is the Diversity, Equity, Inclusion, and Accessibility (DEIA) professional, which was investigated as a potential addition to the portfolio. However, the Personas team learned about the emerging nature of this role in CTSA hubs, making it difficult to develop this profile with confidence currently. For example, we found very few focused full-time DEIA professionals at the CTSA Program hubs to interview. We observed that often the DEIA professional tasks were conducted by an employee with another job title and competing responsibilities or were dispersed within the larger institutional structure. Indeed, one advantage of developing Personas is the identification of gaps within institutional roles. Perhaps as a step toward incorporating dedicated DEIA professionals into CTSA Programs, hubs can consider appointing a Chief Diversity Officer (CDO), or an equivalent leadership position. Recent findings from the commercial and academic sectors highlight the necessity and benefits of appointing CDOs. These findings emphasize that with effective organizational placement, CDOs can positively influence provided services and leverage sufficient funding to support staff, training improvements, and partnership building [15,16]. For example, by examining hiring practices and studying how units operate internally, CDOs can contribute to more equitable workforce recruitment and unit interactions within CTSA hubs [17].

We invite the users of the Persona profiles to share future potential improvements. We encourage users to adapt and tailor CTS Persona profiles at their institutions in order to best serve the needs of their local communities. The flexible, adaptable, and ever-improvable nature of the CTS Personas will hopefully guarantee their usefulness for years to come.
